# Markets, merit and the dignity of labour

**DOI:** 10.1007/s12232-022-00402-7

**Published:** 2022-08-08

**Authors:** Robert Sugden

**Affiliations:** School of Economics, University of East Anglia, Norwich, UK

**Keywords:** Merit, Meritocracy, Dignity of labour, Contributive justice, D63 (Equity, justice, inequality, and other normative criteria and measurement)

## Abstract

In *The Tyranny of Merit*, Sandel recasts the discontent expressed in populism as a rejection of market morality and as an inarticulate plea for the restoration of civic virtue. He argues that a ‘market-based globalisation project’ has fostered meritocratic ideas which humiliate the victims of that project and undermine the dignity of labour. I question Sandel’s claim that meritocracy is a market value and the dignity of labour is not. I argue that his account of a moral alternative to normal market institutions—an economy in which individuals’ rewards are somehow aligned with their true merits—is deeply incoherent.

## Introduction

In June 2016, a majority of British voters chose that their country should leave the European Union. In November 2016, Donald Trump won the US Presidential election on a populist platform. Among the lesser consequences of those two events has been the emergence of a new genre of literature—the genre of *Brexit/Trump anguish*. Books in this genre are written by liberal or left-leaning journalists, political scientists, economists and philosophers. Their common premise is that Brexit and Trump supporters voted for political programmes that were—and that they should have known to be—contrary to their own interests and to the wider interests of their communities and nations. For the authors of such books, two features of these votes are particularly disturbing. First, Brexit and Trump were disproportionately supported by lower-income and less-educated voters—people whose interests left-of-centre political movements would be expected to promote. Second, those voters seemed to have deliberately rejected economic, political and moral positions that were almost unquestioned within the professional and intellectual communities to which the various authors belong. Indeed, a thinly-veiled theme in the Brexit and Trump campaigns had been resentment against (not to say contempt for) claims of expertise and moral rightness made by exactly those communities—the so-called ‘metropolitan elite’. Typically, the author’s response is to offer an explanation of these phenomena that treats the Brexit and Trump votes as misdirected expressions of understandable and at least partly legitimate discontent. Some authors look for ways of adapting the left-liberal consensus to respond to that discontent without compromising what, for them, are its most fundamental principles. In other cases, the argument is that the voters were expressing attitudes which, if properly understood, were in support of positions that the author has already defended and that are not in need of significant revision. In his recent book *The Tyranny of Merit*, Michael Sandel (2020) contributes to this branch of the genre.

Sandel is the author of two previous books which develop moral critiques of markets and of kinds of thinking that, he argues, are propagated by an excessive use of market mechanisms and by economic theories that purport to justify those mechanisms. In *Justice: What’s the Right Thing to Do?*, Sandel (2009: p. 9) argues that justice is about ‘giving people what they deserve’. That requires judgements about ‘what virtues are worthy of honor and reward, and what way of life a good society should promote’. A major theme of that book is that the market generates incomes that are not properly aligned with the virtues of the people who receive them. In *What Money Can’t Buy: The Moral Limits of Markets*, Sandel (2012: pp. 128, 203) argues that markets can corrode morality by fostering ‘market values’ which tend to undermine the civic virtues of solidarity, ‘shar[ing] in a common life’, and ‘car[ing] for the common good’.^1^ In *The Tyranny of Merit*, Sandel tries to recast the discontent of the Brexit and Trump voters as a rejection of what he has characterised as market values and as an inarticulate plea for the restoration of civic virtue.

Luigino Bruni and I (2013) have already presented critiques of Sandel’s 2009 and 2012 discussions of market values. In the present paper, I focus on two new themes that are prominent in *The Tyranny of Merit*. One is the idea of *meritocracy*, which Sandel presents as a market value that underpins the hubris of people who are economically successful and that humiliates those who are not. The other is the *dignity of labour*, which Sandel presents as a civic value that can be undermined by market forces. By making many traditional manufacturing industries unprofitable, globalisation has deprived working-class people of opportunities for meaningful work. According to Sandel’s diagnosis, the grievances that have fuelled populist politics ‘are not only economic but also moral and cultural; they are not only about wages and jobs but also about social esteem’ (Sandel, 2020: p. 18).

Sandel’s primary concern is with these moral and cultural grievances. It should go without saying that any comprehensive explanation of the growth of populism must take account of deep changes in the British and American economies which, independently of any changes in social values, have affected the prospects of working people. These changes include the increasing importance of services relative to manufacturing, developments in digital technology that displace many forms of skilled labour (either directly or by allowing outsourcing to lower-wage countries), the effects of East Asian economic growth on international trade patterns, and (partly as a result of these changes) increasing income inequality. Clearly, these effects are *transmitted* through markets, but whether they are *caused* by markets, and whether they can be attributed to the dominance of market values in political discourse, is much less obvious. Sandel sometimes seems to conflate these three ideas, most obviously in his repeated references to an undefined ‘market-driven project of globalization’ (2020: p. 5, see also pp. 19, 184, 202, 213, 222). This is an example of a more general tendency in Sandel’s view of economics that I will say more about later—a tendency to treat economists’ statements about the facts of economic life as judgements of moral approval (and of moral approval of a very particular kind). For the moment, it is sufficient to say that my paper is a commentary on Sandel’s moral and cultural analysis of market values, and not a discussion of the underlying economic causes of the discontents that fuel populism. I will question Sandel’s claim that meritocracy is a market value and that the dignity of labour is not. I will argue that his account of a moral alternative to ordinary market institutions—a thinly described economy of republican virtue in which individuals’ economic rewards are aligned with their true merits—is deeply incoherent.

## The meritocratic sensibility

Sandel gives a lot of attention to the language of public debate, and particularly to the words of recent US Presidents and presidential candidates (with the notable exception of Donald Trump). He identifies a recurring theme, the ‘rhetoric of rising’, which expresses a ‘meritocratic sensibility’ (p. 61).^2^ Here, are some of his examples^3^:

Ronald Reagan in 1984, speaking to black members of his administration: ‘All Americans have the right to be judged on the sole basis of individual merit and to go just as far as their dreams and hard work will take them.’

Bill Clinton in 1993: ‘The American dream that we were all raised on is a simple but powerful one: if you work hard and play by the rules, you should be given a chance to go as far as your God-given ability will take you.’

Tony Blair in 1996, writing about the programme of ‘New Labour’: ‘We believe that people should be able to rise by their talents, not by their birth or the advantages of privilege’.

Barack Obama in his 2012 presidential re-election campaign: ‘[W]hat makes America so exceptional, what makes us so special, is this basic bargain, this basic idea in this country, no matter what you look like, no matter where you come from, no matter what your last name is, no matter what setbacks you may experience, in this country, if you work hard, if you are willing to take responsibility, then you can make it. You can get ahead.’

Hillary Clinton in her 2016 presidential campaign: ‘Our campaign is about the fundamental belief that, in America, every person, no matter what you look like, who you are, who you love, you should have the chance to go as far as your hard work and dreams can take you.’

Theresa May in 2016, after becoming Prime Minister in the aftermath of the Brexit vote, and speaking about what her government will do for ‘ordinary, working-class people’: ‘I want Britain to be … a country where everyone has a fair chance to go as far as their talent and their hard work will allow. … I want Britain to be a place where advantage is based on merit, not privilege; where it’s your talent and hard work that matter, not where you were born, who your parents were or what your accent sounds like.’

The similarities of phrasing between these quotations may tell us something about how political speechwriters recycle one another’s material, but I think it is fair to assume that these six speakers are expressing genuine political beliefs in language that is intended to resonate with their audiences. Some common themes emerge clearly. The American speakers (but, interestingly, not the British ones) make patriotic appeals to a principle of equality of opportunity that is supposedly part of their audience’s national identity. There is an implicit recognition that, in reality, some people—variously defined by colour, country of origin, sexual orientation and social class—are currently denied full equality of opportunity, but this is presented as an injustice that the speaker is committed to righting. The ‘as far as your—can take you’ clause is filled in with different combinations of ‘merit’, ‘ability’, ‘talent’ and ‘dreams’ (which can perhaps be translated as ambitions or aspirations), always together with ‘hard work’. What exactly is being promised by a commitment to equality of opportunity is not altogether clear; I will discuss some of these ambiguities later. My immediate concern, like Sandel’s, is with the rhetoric. To whom is it likely to appeal?

It is easy to imagine its appeal to a talented individual who is trying to make her way in a competitive career that offers good rewards to those who succeed, but whose efforts to succeed are being held back by unfair discrimination. Think of an aspiring sportsperson, artist, lawyer or academic who finds the cards stacked against her because of her colour, gender, social class or the name of the university in which she was trained. It is easy, too, to imagine its appeal to a recent immigrant from a background of poverty, starting out on a new life in a country where there are disadvantages to be overcome but opportunities to be taken. Leaving aside the issue of discrimination, the rhetoric of rising might appeal to any energetic young person hoping to enter some career that requires learned skills—whether those of an accountant or a hairdresser, a doctor or a truck driver—and that offers opportunities to progress. But, as Sandel rightly points out, these fine words are not so inspiring for a working-class person in a declining industrial region whose efforts to find a job keep meeting with failure. That person’s problem cannot be overcome by working harder. Nor can it be attributed to unfair discrimination against some characteristic that he can assert as his identity. The problem is the state of the market: the skills that he can offer are no longer in demand.

Sandel quotes Arlie Russell Hochschild’s immersive sociological study of the political attitudes of working people in rural Louisiana. Summarising ‘the hopes, fears, pride, shame, resentment, and anxiety’ of the people she talked with, she describes their sense of ‘a struggle to feel seen and honored. And to feel honored you have to feel—and feel seen as—moving forward. But through no fault of your own, and in ways that are hidden, you are slipping backward’ (Hochschild, 2016: pp. 135, 144). For someone in this position, the rhetoric of rising is an additional humiliation. Sandel argues that this rhetoric has helped to fuel the pains and resentments that populist politicians can turn into votes. I think that is right.

But what makes meritocracy a *market* value? Given Sandel’s thesis about market values, it is curious that he devotes two chapters to the competition for admission to elite American universities such as his own Harvard. He is right to treat this competition as a pathology of *meritocracy*, but in what sense is it a pathology of *markets*? Viewed in the perspective of economics, Harvard is not a profit-seeking firm producing for a competitive market. It is a non-profit institution which both creates and trades on the scarcity of its product. By using ultra-selective methods to choose the tiny fraction of eager would-be customers with whom it actually trades, it produces certifications that can be converted into material rewards and social esteem: it is primarily a ‘sorting machine’ rather than a seller of education (Sandel, 2020: p. 177).^4^ In his discussion of elite university admissions, Sandel says almost nothing to link this case study of meritocracy to the moral values of the market. He points out that increases in income inequality are increasing the financial value of the sorting process to successful applicants (p. 12), but that is simply an economic fact. And he describes successful applicants as ‘the winners of a hyper-competitive admissions process that unfolds against the background of a hyper-competitive market society’. The suggestion seems to be that the meritocratic values revealed in the admissions process are induced by, or at least analogous with, moral values induced by markets; but the connection is not explained.

More fundamentally, what is the special link between markets and the rhetoric of rising? Being able to go as far as your dreams, ability and hard work can take you is something you might hope for in any competition in which success is rewarded, whether the reward is in material goods or esteem, and whether the mechanism that allocates rewards is market-based, political (think of winning an election), administrative (promotion in the military or civil service), athletic (Olympic medals) or peer approval (literary prizes or publication of research papers in prestigious journals). It is also something that you might hope for in a career in which workers do not compete with one another at all. Think of a young person whose ambition is to be a truck driver. A confident girl with this ambition might well hope that drivers who show themselves to be hard-working, responsible and resourceful will be able to earn higher-than-average wages and be trusted with more demanding assignments. She would certainly hope that female drivers were not paid less than equally competent men. These hopes do not presuppose that truck drivers compete with one another in the sense that athletes compete in a race, or sales staff compete for a ‘salesperson of the month’ award.

Sandel prefaces his account of the rhetoric of rising with the claim that it originated in the ‘market triumphalism of the 1980s’ and that its sub-text is a meritocratic rationale of the market. Here, is Sandel’s formulation of that supposed rationale: ‘Provided they operate within a fair system of equal opportunity, markets give people what they deserve. As long as everyone has an equal chance to compete, market outcomes reward merit’ (p. 62). But notice that none of six quotations actually mentions the market. Nor, I think, does any of them claim that, if there were equal opportunity to compete, ex post rewards would be aligned with merit.

Bill Clinton, Hillary Clinton and May all talk about a *chance* or *fair chance* to succeed. Blair’s ‘able to rise by their talents’ formula is ambiguous, but ‘being able to’ might reasonably be read as ‘having a fair chance to’. None of those four speakers claims that fair competitions are always won by the most meritorious entrants. Reagan’s notion of an American right to achieve your dreams is surely not intended to be interpreted literally: no form of social organisation could promise that. As I read it, Obama’s ‘American bargain’ is a promise that people who work hard and take responsibility will achieve a decent standard of living and share in the nation’s expected economic growth. Since he cannot be promising that everyone who works hard will get ahead of everyone else, I think he must mean that hard-working people will ‘get ahead’ in the sense of making their way in life. In the same re-election campaign, Obama famously declared that ‘if you were successful, you didn’t get there on your own’: you may think that you are successful because you are smart and hardworking, but ‘there are a lot of smart people out there’ and ‘a whole bunch of hardworking people out there’ who have been less successful than you (quoted by Sandel, p. 131). In other words, the American bargain does *not* promise that rewards will be aligned with talent and effort.

One might have expected Sandel to pick out, and then debate with, philosophical defenders of markets who have actually claimed that market outcomes reward merit. Instead, he debates with Friedrich Hayek, whom he rightly describes as having made ‘[p]erhaps the most influential case for free-market liberalism’ (p. 126). But, as Sandel acknowledges, Hayek explicitly denies that markets reward merit or moral desert. Sandel suggests that this denial is Hayek’s ‘way of fending off demands for redistribution by those who believe that hedge fund managers do not deserve to make more than teachers’ (p. 128). That is unfair: rightly or wrongly, Hayek argues that markets *cannot* reward merit, but that in the long run, everyone can expect to benefit from the wealth that markets create.

Hayek makes a clear distinction between market value (in the normal economic sense of the term) and moral merit. The market value of a good or service is the price it commands in the market. Under conditions of competition, the market value of a person’s labour is what, at the margin, other people are willing to pay for what that labour produces. When Hayek writes about merit and desert, he refers to a person’s efforts and sacrifices, not to what those efforts and sacrifices produce. There is no reason to expect market value to be aligned with that kind of merit, and Hayek does not pretend otherwise. It is fundamental to Hayek’s justification of the market that any system of economic organisation has to face the problem of the *division of knowledge*. If wealth is to be created by coordinated action, there has to be some decentralised system for integrating items of knowledge that are distributed among economic agents. The market solves this problem by rewarding individuals for discovering ways of transacting with other people that those other people find beneficial. The implication is that each individual is rewarded according to the actual benefits that his actions confer on others. That this reward mechanism can treat individuals unfairly is a price that has to be paid for the market’s wealth-creating properties, and is morally analogous with the unfairness of nature:

Are we not all constantly disquieted by watching how unjustly life treats different people and by seeing the deserving suffer and the undeserving prosper? And do we not all have a sense of fitness, and watch it with satisfaction, when we recognize a reward to be appropriate to effort or sacrifice? ... [W]e experience the same feelings also with respect to differences in human fates for which clearly no human agency is responsible and which it would therefore clearly be absurd to call injustice. Yet we do cry out against the injustice when a succession of calamities befalls one family while another steadily prospers, when a meritorious effort is frustrated by some unforeseeable accident, and particularly if of many people whose endeavours seem equally great, some succeed brilliantly while others utterly fail. ... And we will protest against such a fate although we do not know anyone who is to blame for it, or any way in which such disappointments can be prevented. (Hayek, 1976: pp. 68–69)

The unfairness of market rewards can be mitigated by redistributive taxation and by social insurance schemes, but it cannot be eliminated without disabling the mechanisms by which markets work.^5^

Sandel’s response is that Hayek’s ‘renunciation of merit and desert is not as thoroughgoing as may first appear’ because ‘if, as Hayek claims, economic value is a legitimate basis for inequality, it is not so clear that invidious attitudes to success are undercut’ (pp. 134–135). On Hayek’s account, each individual’s rewards in a market economy are aligned with the market value of his marginal contribution to society. Sandel points out that a successful person can still feel hubris about the high market value of his contribution, and an unsuccessful one can still feel humiliated when she is reminded about the low market value of hers. He thinks that this makes the distinction between merit and value ‘vanishingly thin’ (p. 135), and claims that Hayek is uncritically accepting the assumption that ‘a person’s market value is a good measure of his or her contribution to society’ (p. 136). But here Sandel is conflating economic facts with moral values. That a person’s wage is a measure of the *market* value of the marginal product of her labour, expressed in terms of the prices that the buyers of that product pay, is an empirical proposition; it describes a broad tendency of competitive labour markets. Hayek is not responsible for that fact, and he has done his best to explain that market value is not moral merit. What more can Sandel ask of him? It may well be a fact of human psychology that people who are successful in life tend to believe that this is a deserved reward for their merits, but if that *is* a fact, it surely applies to all forms of success, not only to success in the market. In any case, Hayek is not responsible for human psychology.

At the most fundamental level, it is not Hayek, Reagan, Bill Clinton, Blair, Obama, Hillary Clinton or May who is the advocate of meritocracy; it is Sandel. It is Sandel who maintains that society should reward people according to their moral merits. His criticism of the market is that it does not reward what he judges to be moral merit. To complete his argument, he needs to show that the working-class Brexit and Trump voters were expressing something like the same criticism. This is where the dignity of work comes in.

## The dignity of labour

Following Anne Case and Angus Deaton (2020), Sandel describes the economic decline of American working-class communities as the loss of a way of life. Unemployment and economic insecurity has led to reductions in life expectancy that are largely due to suicides, drug overdoses and alcoholism. Working-class people are ‘giving up on work’ and even ‘giving up on life itself’. Sandel attributes much of this despair to an ‘insidious injury’ inflicted by meritocratic and neo-liberal thinking—an erosion of the dignity of work (pp. 198–202).

In explaining his understanding of the dignity of work, Sandel draws on Catholic social thought, and particularly on the concept of *contributive justice*, as formulated in a pastoral letter of the United States Conference of Catholic Bishops in 1986:

*Social justice implies that persons have an obligation to be active and productive participants in the life of society and that society has a duty to enable them to participate in this way.* This form of justice can also be called ‘contributive’, for it stresses the duty of all who are able to help create the goods, services, and other nonmaterial or spiritual values necessary for the welfare of the whole community. … The meaning of social justice also includes a duty to organize economic and social institutions so that people can contribute to society in ways that respect their freedom and the dignity of their labor. … Work with adequate pay for all who seek it is the primary means for achieving basic justice in our society. (pp. 17–18, italics in original)

As summarised by Sandel (p. 212), this concept of justice is grounded on the idea that ‘the fundamental human need is to be needed by those with whom we share a common life. The dignity of work consists in exercising our abilities to answer such needs’. Work is meaningful when it can be seen as a person’s contribution to the common good of society, for which his or her wage is a return. As expressions of this idea, Sandel (pp. 210, 212) quotes two political speeches from 1968. One is by Robert Kennedy, saying that essential values of civilisation come from ‘dignified employment at decent pay, the kind of employment that lets a man say to his community, to his family, to his country, and most important, to himself, “I helped build this country”. I am a participant in its great public ventures’. The other is by Martin Luther King to striking sanitation workers in Memphis: ‘One day our society will come to respect the sanitation workers if it is to survive, for the person who picks up our garbage is in the final analysis as significant as the physician, for if he doesn’t do his job, diseases are rampant. All labor has dignity.’

The core idea behind these statements is that the dignity of labour is a form of mutual respect, based on people’s recognition of one another as contributors to a common enterprise, and that sharing in the dignity of labour is an important form of self-respect. That seems right. It helps to explain the demoralisation of the job-seeking unemployed and of people whose previously marketable skills are made obsolete, and the humiliating effect of the rhetoric of rising. But when Sandel explains how he understands the common enterprise that gives labour its dignity, his argument loses track.

Sandel treats ‘production’ and ‘consumption’ as distinct moral domains. Production is presented as a domain of civic virtue, and consumption as a domain of market values. The never-defined project of globalisation ‘focus[es] on maximizing GDP’ and ‘invites us to think of ourselves more as consumers than as producers’. When we think as consumers, we think in a morally impoverished way: ‘[W]e want to get the most for our money, to buy goods and services as cheaply as possible, whether they are made by low-wage workers overseas or well-paid American workers’ (p. 207). But: ‘From the standpoint of the civic conception, the most important role we play in the economy is not as consumers but as producers. For it is as producers that we develop and exercise our abilities to provide goods and services that fulfil the needs of our fellow citizens and win social esteem.’ What is special about production is that ‘work draws citizens together in a scheme of contribution and mutual recognition’. Taking this perspective, Sandel rejects Adam Smith’s dictum—one that most economists of all periods would probably endorse—that consumption is the sole end and purpose of all production (p. 209).

Sandel is not seeing the fundamental complementarity of production and consumption. He is right to say that the meaningfulness of production—the feature that distinguishes it from play—is that it creates things that, when considered without reference to the production process itself, fulfil people’s needs. Thus, one facet of the dignity of labour is the recognition that the worker’s labour is needed by others. But the route by which the other people’s needs are fulfilled is through their consumption of the product of that labour. However many intermediaries there may be, paid work in a market economy is ultimately a relationship of exchange between people in the role of workers and people in the role of consumers. The basis for mutual respect is that each party to this relationship benefits from the participation of the other. As a producer, the worker gains dignity from the knowledge that he is creating something that other people will value as consumption. Thus, the steelworker can take pride in the bridges that other people want to cross; the sanitation worker can take pride in the cleanliness that protects other people against sickness. In a market economy, the worker’s wage is an acknowledgement that he has created something that has value to someone else.

Consumption enters into the dignity of labour in another way—one that may be even more important for self-respect. A worker’s wage is the means by which he is able to consume the products of other people’s labour. As a consumer, he gains self-respect from the knowledge that he is able to pay for what he consumes, and that the money he pays has been earned by his own labour. When he shops at a supermarket, he can engage with its owners on terms of equality: he wants what they sell, but they want his custom. That mutually beneficial market relationships support this kind of self-respect is an important part of Adam Smith’s (1776/1976: p. 45) famous account of how we get our dinners without having to appeal to anyone’s benevolence.^6^ Contrast the position of someone who, because of unemployment or insufficient wage income, has to make use of a charitable food bank. However well-intentioned such a charity may be, there is a humiliation in being dependent on it. In traditional working-class communities, being a ‘good provider’—a man who could reliably provide his family with a decent standard of living—was one of the cardinal virtues of a husband and father. Sandel gives surprisingly little attention to this aspect of the dignity of labour—perhaps because, for his taste, it is too closely connected to consumption.

Sandel’s morally dismissive attitude to consumption is grounded in his ‘civic’ account of the common good. He links the distinction between consumption and production to a distinction between two ways of understanding the common good. The ‘consumerist’ understanding ‘defines the common good as the sum of everyone’s preferences and interests’. In contrast, the civic understanding ‘is about reflecting critically on our preferences—ideally, elevating and improving them—so that we can live worthwhile and flourishing lives…. It requires deliberating with our fellow citizens about how to bring about a just and good society, one that cultivates civic virtue and enables us to reason together about the purposes worthy of our political community’ (pp. 208–209). For Sandel, then, what is objectionable about the consumerist understanding of the common good is that it factors the common good into the separate good of each individual, and allows each individual to make her own judgements about her own good without being required to deliberate with anyone else about the worthiness of those judgements. On the consumerist view, the common good of people who interact with one another is whatever is common to their several conceptions of their good as individuals. Since this way of thinking about the common good has no special connection with consumption, ‘consumerist’ seems an inappropriate label. A better word might be ‘liberal’.

Sandel’s main concern is not with the value of the common good itself, but with assessing the value of individual contributions to it. The underlying idea is that a person’s merit as a producer derives from the value of her contribution to the common good, and that labour derives its dignity from a social recognition of that merit. Sandel is looking for collective judgements that compare the ‘true value’ of different people’s contributions, and thereby assign degrees of merit. His objection to ‘consumerism’ is that it produces what he believes to be unacceptable judgements of this kind: ‘If the common good is simply a matter of satisfying consumer preferences, then market wages are a good measure of who has contributed what. Those who make the most money have presumably made the most valuable contribution to the common good, by producing the goods and services that consumers want’ (p. 208). In response, he says: ‘The true value of our contribution cannot be measured by the wage we receive… [It] depends instead on the moral and civic importance of the ends our efforts serve’ (p. 209). What is to count as a ‘truly valuable contribution’ is a matter for democratic deliberation within a political community (p. 214).

But what exactly are citizens to deliberate about, and why? Sandel does not give any serious consideration to the kinds of arguments that might be advanced in this deliberation, or to the kinds of conclusions to which it might lead. He relies on simplistic claims about true value which, it seems, he expects his readers to find so obviously right that they will see no need to ask difficult questions about what these claims actually mean. For example, and with implicit approval: ‘Some people argue that hedge fund managers do not deserve to make vastly more money than schoolteachers; managing money is far less admirable and important than teaching and inspiring young people’. He adds the thought that a ‘defender of free markets’ might argue that hedge fund managers’ work on investing ‘the hard-earned pensions of teachers, firefighters, and [perhaps less convincingly] college endowments’ is admirable and important too, but implies that this consideration would not justify the ‘vast sums’ that they earn (p. 127). Or, comparing a casino mogul who ‘makes thousands of times more than a nurse or a doctor’: ‘Caring for people’s health is morally more important than catering to their desire to play slot machines’ (p. 139).

An even more striking omission is that Sandel does not tell us the purpose of this deliberation. From the requirement that the deliberation is to be *democratic*, it is natural to infer that its purpose is to reach collective decisions, and not merely to allow differing judgements to be expressed and debated. But collective decisions about what? Although Sandel never says this outright, I think the idea must be that the decisions are about the distribution of rewards, and that the aim is to increase the alignment of individuals’ rewards with their contributions to the common good, thereby conferring greater dignity on truly productive labour. For example, in his discussion of the dignity of labour, Sandel refers with apparent approval to Émile Durkheim’s argument that ‘the division of labor can be a source of social solidarity, provided everyone’s contribution is remunerated according to its real value to the community’ (p. 211). In his very brief discussion of concrete policies to uphold the dignity of work, he approves of tax changes that are designed to express society’s judgements about ‘what counts as a valuable contribution to the common good’ and ‘which activities are worthy of honour and recognition’ (pp. 218–219).

But the idea of measuring the ‘true value’ of a person’s contribution to a morally and civically defined common good is deeply problematic, and it is not at all clear that attempts to align wages with contributions to true value would support the dignity of labour. Here are a few of the difficulties.

*Diamonds and water*. Many early economists struggled with the diamonds–water paradox: Why is the price of diamonds so high and the price of water so low, when water is so much more important for human life than diamonds? The paradox dissolves if one distinguishes between *marginal* and *total* value (or utility). Providing health care is undoubtedly more important than providing slot machines in the same sense that water is more valuable than diamonds, but is the product of a marginal day of work in a hospital more important than the product of a marginal day of the same type of work in a casino? In the UK, most health care is supplied free of charge by the state-run National Health Service. The proportion of national income devoted to health care—in other words, the allocation of resources between socialised health care and private consumption—is the subject of constant public debate and is determined by democratic political processes, not market ones. Can Sandel really be sure that an additional £1 devoted to the National Health Service would have more civic value than an additional £1 devoted to individuals’ chosen forms of private consumption (which, for some people, might be playing slot machines)?

*The two cleaners*. Suppose we accept Sandel’s claim that, measured in terms of true value, the marginal product of labour is higher in the health care industry than in the gambling industry. Think about Rosie, whose job is to clean the public areas of a hospital, and Sally, whose job is to clean the public areas of a casino hotel. If health care really is far more valuable and admirable than slot machines, Rosie’s contribution to the common good must be far more valuable and admirable than Sally’s. But how are we supposed to imagine an economy in which jobs with essentially the same skill requirements and working conditions are paid differently, according to a collective judgement about the civic importance of the ends that are being served? Leaving aside the question of wage differences, how does it support the dignity of Sally’s labour to tell her that, although she is just as good a cleaner as Rosie, her work is not as admirable?

*The Uber driver*. Think about Ahmed, who is an Uber driver. His work is driving customers from place to place. He knows that *they* value his services, because they ask him to carry them and pay him for doing so, and he can take pride in being a careful and knowledgeable driver without enquiring further into the purpose of their journeys. But, according to Sandel, the true value of Ahmed’s contribution to the common good depends on the moral and civic importance of the ends that those journeys serve. His labour has more dignity if he drives Rosie to her workplace than if he drives Sally to hers. Should he be paid more when Rosie is the customer, and if so, to whom should this extra payment be charged? (Not Rosie, I hope.) And leaving aside the question of payment, how does it support the dignity of Ahmed’s labour to tell him that taking Sally to where she wants to go is less admirable than taking Rosie to where she wants to go? Notice also that, even in this simple case, we are coming up against the problem of the division of knowledge: in a complex economy, individuals cannot know the ultimate ends that are served by their economic activities. In many cases, Ahmed cannot know what ultimate ends are served by his customers’ journeys: the fact that the customers are willing to pay him is the only certification he has that needs are being satisfied.^7^

*Middlesbrough and the Sydney Harbour Bridge.* I was brought up in Middlesbrough, the principal town in an industrial area in north-east England. From the middle of the nineteenth century, Middlesbrough had grown from nothing to a become a major centre of iron and steel production and heavy engineering; it had chosen the splendid motto ‘Erimus’ (We Shall Be). When I was young, Middlesbrough was still relatively prosperous, and we took pride in our heavy industries. (In our *plein air* art lessons at school, we sketched smoking blast furnaces.) A particular source of local pride was that a Middlesbrough firm had built the Sydney Harbour Bridge. Since then, Middlesbrough’s main industries have collapsed, and the area is now one of the most deprived in the UK. As in the Rust Belt communities of the US, there has undoubtedly been a loss of the dignity of labour that Robert Kennedy expressed in the phrase ‘I helped build this country’. But remember that building the Sydney Harbour Bridge was not part of the enterprise of building *our* country: the pride came from the fact that Middlesbrough had been chosen to build one of the greatest bridges in the world. The moral value that the Middlesbrough workers had created was not ‘civic’ in Sandel’s political sense: it was conferred by the judgements of Australians. The point of this example is that the common good to which labour contributes need not be the good of the political community to which the worker belongs. This is not something that began with the ‘globalisation project’ of the 1980s, or even with the Industrial Revolution: economic transactions that take place across the boundaries of political communities are older than recorded history. And this is a two-way street. If the dignity of the labour of Middlesbrough steelworkers in the 1920s came from meeting the needs of Australian rail travellers, the American consumers who buy imported goods in the 2020s are supporting the dignity of workers in other countries. The protectionism that Sandel seems to support is not just protectionism in the economy of goods, it is protectionism in the economy of dignity.

*Bill Gates and his gardener*. In his response to Hayek, Sandel argues that a person whose labour has a low market value can feel humiliated when she compares her wage with the income of someone whose labour has far higher market value. He chooses examples in which he thinks it is obvious that the difference in market value does not correspond with a difference in the true value of their contributions—for example, the casino mogul who earns thousands of times more than the nurse. But if we are serious about measuring the value of individual contributions to the common good, there are many cases in which, on any reasonable measurement of ‘true value’, some individuals really do contribute far more than others. Think of Bill Gates and a person (say Joe) whom he employs to work in his garden. Joe’s work creates a very small addition to the total consumption of someone who is extremely rich. Even leaving aside the immense value of Gates’s philanthropy, it is entirely credible to think that Gates’s contribution to the common good is thousands of times greater than his gardener’s. So if large differences in the true value of individuals’ contributions are humiliating to those whose contributions are small, we have a problem that has no solution in a modern economy. But how does the difference between the two men’s contributions to the common good undermine the dignity of Joe’s labour? That difference has no bearing on the economic relationship between Bill and Joe. Like Adam Smith’s shopkeeper and his customers, Bill and Joe can interact with mutual respect. Bill values the tidiness of his garden; Joe values the income he earns by his work. Bill and Joe have a common understanding that their intention in interacting is to achieve the goodness of a combination of outcomes (the tidiness of Bill’s garden, the consumption that is paid for by Joe’s wages) that is common to their separate conceptions of individual good.^8^

## Implications

If there is any truth in the arguments I have presented, it is fanciful to suppose that, in a complex modern economy, rewards can be aligned with a credible civic conception of merit. Sandel avoids facing up to this reality by devoting less than eight of his 227 pages to concrete policy proposals, and by restricting himself to three proposals that are only tangentially related to the project of rewarding civic merit (pp. 214–221).

Sandel’s first proposal is a wage subsidy for low-income workers, thus ‘enabling low-income workers to make a decent living even if they lack the skills to command a substantial market wage’ (p. 214). This proposal has an obvious justification on grounds of contributive justice: recall the US Catholic Bishops’ declaration that a government has a duty to organize economic and social institutions so that people can contribute to society in ways that respect their freedom and the dignity of their labour. A wage subsidy is an effective way of ensuring that people who are able to work can achieve a minimum standard of living through productive labour. But if the real aim is to align rewards with civic merit, this is a crude instrument. For example, if the market wage for cleaners were sufficiently low, this policy would give the same subsidy to Sally as it does to Rosie. Provided that one accepts that cleaning hospitals and cleaning casinos are both positive contributions to society, contributive justice would be served, but merits (as judged by Sandel) would not be given their proper rewards.

However, this proposal is hardly new. A wage subsidy is essentially the same as a tax credit system, such as those that already operate in the US and the UK. The US Earned Income Tax Credit was introduced under the presidency of Gerald Ford, significantly expanded under Reagan (who was an enthusiastic supporter), and expanded again under Bill Clinton. The UK tax credit system was introduced by Margaret Thatcher’s government and extended by Blair’s. The Blair government also introduced a complementary policy with a similar objective—a legally enforced minimum wage.^9^ The names of these Presidents and Prime Ministers are significant. In Sandel’s ideological classification scheme, Thatcher and Reagan are the co-progenitors of the globalisation project, and Blair and Clinton (with Gerhard Schröder) are the centre-left politicians who ‘moderated but consolidated the market faith’ (pp. 20–21). There is no evidence here of a fundamental conflict between the globalisation project (whatever that may be) and contributive justice.

Sandel’s other proposals are to equalise the tax rates on capital gains and earned income, and to impose a tax on financial transactions. These proposals are presented as contributions to a ‘public debate about what counts as a valuable contribution to the public good’. Sandel interprets the current low tax rate on capital gains and the absence of taxes on many forms of financial transaction as expressing a civic judgement that investors are more deserving of honour than workers—a judgement that affronts the dignity of labour and should be reversed (pp. 219–221). However, the same proposals can be justified without making any judgements about the relationship between merit and reward. Most economists would probably support the equalisation of tax rates on different forms of income on grounds of efficiency, fairness and the reduction of tax evasion. There is less agreement about the advantages and disadvantages of a financial transaction tax, but ever since Tobin’s (1978) original proposal for a tax on foreign exchange transactions, economic debate has focused on the effects of such taxes on the efficiency and volatility of markets.^10^

It is natural to ask why Sandel focuses on such indirect policies for achieving his civic objectives. An immediate answer might be that he is a moral philosopher, not an economist. Reasonably, he is sifting through proposals that are already on the political agenda, looking for those that are most in accord with the moral principles he upholds. But that would still leave the question of why he has been unable to find practical proposals that aim directly at rewarding merit and that would thereby restore some of the dignity of labour that the globalisation project has supposedly undermined. Perhaps no practical proposals have been put forward because what Sandel is looking for is not a practical proposition.

My own conclusion is that Sandel’s project of aligning economic rewards with civic merit is a blind alley. I return to the quotations from Ronald Reagan, Bill Clinton, Tony Blair, Barack Obama, Hillary Clinton and Theresa May that Sandel sees as meritocratic, and to those from Robert Kennedy, Martin Luther King and the Catholic bishops that he sees as expressing civic solidarity. To different degrees, they appeal to three fundamental principles that are orthogonal to Sandel’s project. The first is a principle of *fair chances*: the economic opportunities open to an individual should not depend on such personal characteristics as gender, ethnicity, social class, religion or sexual orientation, but may depend on native ability. The second is a principle of *contributive justice*: for every individual who is able to work, there should be some way for him to support a decent standard of living through productive work. The third is a principle of *social insurance*: for every individual, whatever her past choices and even if she is unable to work, there should be some way for her to achieve a decent standard of living. This combination of principles can be built on different moral foundations—for example, on a concept of mutually beneficial cooperation (Sugden, 2018) or of relational (or democratic) equality (Anderson, 1999). If we think in these terms, we can respect one another’s contributions to the overall scheme of economic cooperation without trying to rank them on a scale of merit.

## Footnotes

1 As Sandel (2012: 208, note 18) acknowledges in a footnote, many of the arguments in that book are similar to ones previously made by Elizabeth Anderson (1993).

2 From here on, unattributed page references are to Sandel (2020).

3 As quoted by Sandel (pp. 67–71). Sandel gives full citations in his footnotes.

4 In passing, I must commend Sandel’s proposal for reform of this admission system – an initial sorting by merit which picks a shortlist of well-qualified applicants, followed by a lottery to pick which shortlisted applicants are admitted. This system would give elite universities stronger incentives to compete on the quality of their product, rather than on the restrictiveness of their entry requirements.

5 For a defence of this reading of Hayek, see Sugden (1993, 2004). Sugden and Wang (2020) develop the concept of a ‘Hayekian procedure’ – a rule-governed procedure in which interactions between individuals can produce consequences that were not deliberately chosen by any individual participant. Markets are paradigm cases. The outcomes of such procedures cannot be expected to conform to pre-determined principles of fairness.

6 For more on this reading of Smith, see Bruni and Sugden (2013).

7 As Bernard Mandeville (1714, Remark G) noticed, the socially recognised merit or demerit of an action dissipates as its effects ripple through an economy. In his example, the Pick-pocket’s stolen money becomes more respectable as it passes successively to the Victualler (i.e. innkeeper), the Wealthy Brewer’s Trusty Out-Clerk (i.e. the brewer’s agent who deals with innkeepers), and the Wealthy Brewer himself.

8 The idea that market relations can express intentions for mutual benefit is developed by Bruni and Sugden (2013), Sugden (2018) and Bruni and Santori (2021).

9 Family Credit was introduced in 1986. It was further developed as Working Families Tax Credit (from 1999) and Working Tax Credit (from 2003). The National Minimum Wage was introduced in 1998. The origins of the US tax credit system are described by Moffitt (2003).

10 For a review of the relevant arguments and evidence, see Matheson (2011).
